# Discovery of Bioactive Properties in the Seagrass *Ruppia maritima* Extract: A Triplatform Assessment of Antioxidant, Antidiabetic, Antibacterial, Cytotoxic, and Analgesic Activities

**DOI:** 10.1155/tswj/5452807

**Published:** 2025-11-26

**Authors:** Inun Nahar Payel, Md. Tanvir Chowdhury, Md. Safayat Hossen Momen, Qurratul Ain Sadia, Nazmul Hasan Eshaque, Md. Jahirul Islam Mamun, Suman Das, Fatema Tuz Zohra, Md. Jakaria Parvez, S. M. Moazzem Hossen

**Affiliations:** ^1^Department of Pharmacy, Faculty of Biological Sciences, University of Chittagong, Chittagong, Bangladesh; ^2^Bangladesh Council of Scientific and Industrial Research (BCSIR), Chattogram Laboratories, Chattogram, Bangladesh; ^3^Department of Pharmacy, North South University, Dhaka, Bangladesh; ^4^Department of Pharmacy, University of Development Alternative, Dhaka, Bangladesh

**Keywords:** analgesic, antibacterial, antidiabetic, antioxidant, cytotoxic, molecular docking, *Ruppia maritima*

## Abstract

*Ruppia maritima*, a seagrass, was investigated for its potential antioxidant, antidiabetic, antibacterial, cytotoxic, and analgesic activities. The acetone extract of its leaves (AERM) was studied using in vitro, in vivo, and in silico methods. To determine its secondary metabolites, the total phenolic and flavonoid content was measured. Antioxidant capacity was evaluated using DPPH and ABTS radical scavenging assays, while antidiabetic potential was assessed through the alpha-amylase inhibition assay. The disc diffusion method was used to examine antibacterial effects, and cytotoxicity was determined using both the brine shrimp lethality assay and the MTT assay on HeLa cells. Analgesic activity was tested via the acetic acid–induced writhing test and formalin-induced paw licking test. Quantitative analysis revealed that AERM contained 19.04 ± 1.91 mg/g of phenolics and 14.71 ± 1.09 mg/g of flavonoids. The extract demonstrated antioxidant activity with IC₅₀ values of 87.92 *μ*g/mL (DPPH) and 209.75 *μ*g/mL (ABTS). Antidiabetic testing indicated an IC₅₀ of 132.05 *μ*g/mL, likely due to *α*-amylase inhibition. Strong antibacterial effects were observed, with efficacy comparable to the standard drug pefloxacin. In cytotoxicity assays, AERM showed an LC₅₀ of 31.41 *μ*g/mL in brine shrimp and a dose-dependent reduction in HeLa cell viability. At doses of 200 and 400 mg/kg, AERM produced significant analgesic effects (*p* < 0.001) in both the acetic acid and formalin-induced pain models. Molecular docking and ADME/T analyses suggested high binding affinities, good pharmacokinetic properties, and a nontoxic profile. Overall, the results indicate that AERM has promising pharmacological potential as a natural therapeutic agent. However, further studies with larger sample sizes, repeated trials, and broader dose-response evaluations in various animal models are essential to confirm these findings.

## 1. Introduction

Medicinal plants are important sources of drugs, with around 80% of people using them in the provision of health care. Many are exploring natural substances instead of synthetic compounds in response to the residual side effects. The admirable antioxidant capacity of medicinal plants and their phytoconstituents is recognized for their role in protecting health from numerous diseases by decelerating oxidation and inhibiting oxidative chain reactions [1]. Antioxidants prevent undesirable changes in food flavor and nutritional value, as well as tissue injury in inflammation, cancer, and atherosclerosis. Therefore, a worldwide effort is being made to discover natural antioxidants with low levels of side effects and appropriate for preventive medicine and food [2]. Diabetes mellitus is a serious cause of mortality and a metabolic disorder characterized by hyperglycemia as a result of inadequate insulin production or poor insulin response [1]. There has been a growing interest in the use of herbal therapies because of the side effects of oral hypoglycemic agents, and now traditional herbal medicine from plants is being used for diabetes management as a potential hypoglycemic agent [3].

Antibacterial agents kill or inhibit microorganisms and differ in their chemical structure and activity against Gram-positive and Gram-negative bacteria. The health benefits of antibacterial agents are questioned due to their possible toxicity and since their use is detrimental to normal flora [4]. Furthermore, pathogenic microorganisms have developed resistance to synthetic antibiotics, which is why there is more interest in using biologically active compounds from neuropharmacological traditional medicinal plants [5]. Cancer is a global health concern characterized by significant morbidity and mortality [6]. It represents the uncontrolled proliferation of atypical cells. Marine cyanobacteria indicate potential toxic activity against brine shrimp and cancer cell lines and are known to produce diverse bioactive metabolites. The brine shrimp lethality bioassay is simple, inexpensive, and widely recognized for assessing toxicological and pharmacological activity and is recommended for toxicity studies on plant extracts and screening toxic cyanobacteria [7].

Pain is a complicated phenomenon with wide variations in its definition, pathophysiological causes, duration, and severity, making it difficult to characterize properly [8]. To fail to relieve pain is both morally and ethically unacceptable. Some current pain medications include opioids and nonopioids, and local anesthetics for local effect, nonsteroidal anti-inflammatory drugs (NSAIDs), and corticosteroids are also used for inflammatory conditions. Still, each of these drugs has the potential for toxicity. There is a continuing need for newly discovered therapeutic agents with effective properties and limited side effects [9].

Globally distributed, *Ruppia maritima* is a submerged angiosperm that seems to flourish in various environments [10]. The capacity to osmoregulate is one of the reasons why *R. maritima L.* (widgeon grass) can withstand a wider range of salinities than any other submerged angiosperm [11]. Usually found in brackish or saline streams, it is seldom observed in marine environments. It is also present in diluted fresh water and water with a high salinity. *R. maritima* can occasionally be found close to *Zostera noltii* populations [12]. *R. maritima* is a submerged monocot angiosperm that is found all over the world and is a member of the Alismatales order. Each year, it produces a significant quantity of biomass in the form of seeds rapidly generated in the rhizomes, roots, and leaves. Because of their quick development and spread, seagrass meadows are among the best submerged aquatic plants for protecting species, particularly in salty environments [13].

There seem to be no studies exploring the pharmacological activities of *R. maritima* extract or its phytoconstituents. Thus, the present study is set to explore the in vitro antioxidant, antidiabetic, and antibacterial properties of *R. maritima* against two Gram-positive bacteria, *Bacillus subtilis* and *Staphylococcus aureus*, as well as three Gram-negative bacteria, *Pseudomonas aeruginosa*, *Escherichia coli*, and *Salmonella* Typhi. Additionally, it will assess the cytotoxic effects and in vivo analgesic activity of the acetone extract from this seagrass. We will also dive into molecular docking and absorption, distribution, metabolism, excretion, and toxicity (ADME/T) analysis to uncover the potential mechanisms of action and pharmacokinetic characteristics of the bioactive compounds found in *R. maritima*.

## 2. Materials and Methods

### 2.1. Drugs and Chemicals

In this experiment, analytical grade chemicals and solvents were used. Morphine sulfate, diclofenac sodium, and pefloxacin were purchased from Square Pharmaceuticals Limited, a Bangladeshi pharmaceutical company. The remaining chemicals had been supplied by the Department of Pharmacy of the University of Chittagong, Chattogram, Bangladesh.

### 2.2. Experimental Animal

Male and female albino rats weighing between 20 and 25 g and 4–5 weeks of age were obtained from BCSIR in Chattogram, Bangladesh. Before starting the experiment, they were placed at normal room temperature for a week to get acquainted with their surroundings. The Swiss albino mice were given tap water and healthy food throughout the experiment.

### 2.3. Animal Euthanasia

The animals were euthanized by the 2013 edition of the guidelines for animal euthanasia and were treated by the values of the Swiss Academy of Sciences and the Swiss Academy of Medical Sciences [14]. The study's design and experimental procedures were reviewed and approved by the Animal Ethics Review Board (AERB) of the Faculty of Biological Science, University of Chittagong, and received ethical clearance under the Number AERB-FBSCU-20250107-(2).

### 2.4. Collection and Identification of the Plant

The *R. maritima* seagrass was collected in August 2023 from Kutubdia, Cox's Bazar, Bangladesh. Mr. Mohammad Forkanul Hamid, Department of Fisheries, University of Chittagong identified it. A systematic herbarium was prepared to facilitate authenticity and future correspondence. Future use of a voucher specimen (2023/004//CU/DP) is preserved.

### 2.5. Preparation of the Plant Extract

To ensure efficient extraction, the collected leaves were ground into powder. The granules were put into a sterile glass container to soak in solvents. For 14 days, the sealed vessel was kept in place with sporadic shaking. The mixture was filtered using Whatman Filter Paper No. 1, and the solvents were evaporated using a rotary evaporator. The blackish crude extracts were collected and stored in the refrigerator for later use. Acetone was used as a solvent to create the acetone extract of *R. maritima*.

### 2.6. Phytochemical Screening

The active ingredients in the crude acetonic extract of *R. maritima* were qualitatively determined using standard procedures [15, 16].

### 2.7. Antioxidant Activity

#### 2.7.1. ABTS Radical Scavenging Assay

The methanolic extract's ABTS radical scavenging assay was performed using Ismail et al. [17] methodology. To put it briefly, distilled water was combined with 2 mM ABTS diammonium salt and 3.5 mM potassium persulfate, which were then left in the dark for 16 h. The extract (100–500 mg/mL) was then combined with 290 mL of ABTS solution and incubated in a 96-well plate for 10 min. At 750 nm, the absorbance was measured. As a positive control, ascorbate was employed. The formula used to determine the ABTS radical scavenging percentage (%) was as follows:

 $$\begin{array}{c} \text{ABTS Scavenging }\left(\%\right)=\frac{\text{Ac}-\text{As}}{\text{Ac}}\times 100, \end{array}$$

where Ac and As stand for the absorbance of the control and extract, respectively.

The extract concentration that caused 50% ABTS radical scavenging was used to get the IC_50_ value.

#### 2.7.2. 2,2-Diphenyl-1-Picrylhydrazyl (DPPH) Scavenging Assay

A modified version of Tshibangu et al.'s [18] methodology was used to perform the DPPH scavenging assay on the methanolic extract. A stock solution containing 0.1 mM DPPH was made in methanol, and 1 mL of the extract (100–500 mg/mL) was combined with 5 mL. A 100–500 mg/mL of ascorbic acid was used as a standard. At 517 nm, the samples' absorbance was measured after being incubated in the dark for 30 min. The following formula, where Ac and As stand for the absorbance of the control and extract, respectively, was used to compute the DPPH radical scavenging.

 $$\begin{array}{c} \text{DPPH Scavenging }\left(\%\right)=\frac{\text{Ac}-\text{As}}{\text{Ac}}\times 100. \end{array}$$

The concentration of extract that caused 50% DPPH radical scavenging was used to get the IC_50_ value.

### 2.8. Antidiabetic Activity

#### 2.8.1. Alpha-Amylase Inhibition Assay

A modified version of Wan et al.'s [19] methodology was used to examine an amylase's inhibitory activity. Acarbose and methanolic extract (20–100 mg/mL) were combined with 10 mL of amylase, which had been preincubated in 20 mM sodium phosphate buffer (pH 6.7), and then incubated for 5 min at 37°C. The reaction mixture was filled to a total volume of 2 mL with the starch solution (0.2% *w*/*v*), and it was then incubated for 5 min at 37°C. Following incubation, 1 mL of the dinitrosalicylic acid reagent was added and placed in a boiling water bath. Deionized water was added after the solution had cooled for 5 min. At 540 nm, the absorbance was measured, and the *α*-amylase inhibition was computed as follows:

 $$\begin{array}{c} \text{Alpha}‐\text{amylase inhibition }\left(\%\right)=\frac{\text{Ac}-\text{As}}{\text{Ac}}\times 100, \end{array}$$

where Ac and As stand for the absorbance of the control and extract, respectively.

The extract concentration inhibiting *α*-amylase by 50% is the IC_50_ value.

### 2.9. Antibacterial Activity

The disc diffusion approach was used for antimicrobial screening [20]. Gram-negative bacteria like *Escherichia coli*, *Pseudomonas aeruginosa*, and *Salmonella* Typhi, as well as Gram-positive bacteria like *Bacillus subtilis* and methicillin-resistant *Staphylococcus aureus* (MRSA), were obtained from the Department of Microbiology, University of Chittagong, and utilized as indicator pathogens. Mueller–Hinton broth (HiMedia) medium was used to subculture indicator bacteria for the entire night at 37°C. After preparation, Mueller–Hilton agar medium (HiMedia) was aseptically transferred into the sterile Petri dishes. A cork borer was also used to create wells and smear the bacterial pathogens onto the agar plates. After adding various quantities of the extract (20, 50, and 80 mg/mL) and the common antibiotic (pefloxacin 30 mg) to the corresponding wells, the plates were incubated for 48 h at 37°C. Following the necessary incubation period, the zone of inhibition was measured in millimeters.

### 2.10. Cytotoxicity Activity

#### 2.10.1. Cell Line Used

The National Centre for Cell Science in Pune provided the human cervical cancer cell line (HeLa), which was cultured in Eagle's minimum essential medium (MEM) supplemented with 10% fetal bovine serum (FBS). The Research Resource Identifier (RRID) for the HeLa cell line was CVCL_0030. Every cell was kept at 37°C, 95% air, 5% CO_2_, and 100% relative humidity. Maintenance cultures were passed every week, and the culture medium was replaced twice a week.

#### 2.10.2. MTT Assay

After being seeded on a 96-well plate with MEM supplemented with 10% FBS, HeLa cells with a cell density of 2104 cells were cultivated for cell attachment throughout the night. Following cell attachment, 200 mL of new MEM containing varying amounts of acetone extract (5–100 mg/mL) was added to the MEM. Fifty milliliters of 3-(4,5-dimethylthiazol-2-yl)-2,5 2,5-diphenyl tetrazolium bromide (MTT) solution (5 mg/mL prepared in PBS) was added to each tube after 24 h of incubation, and the wells were then maintained for 4 h in a CO_2_ incubator. After discarding the MTT-containing media, 200 *μ*L of dimethylsulfoxide (DMSO) was added to each well, and the mixture was left to incubate for 20 min. Following incubation, an ELISA reader was used to read the absorbance at 570 nm [21].

The following formula was used to determine the cells' viability:

 $$\begin{array}{c} \text{Cell viability }\left(\%\right)=\frac{\text{As}}{\text{Ac}}\times 100, \end{array}$$

where As and Ac stand for treated and control cell absorbance, respectively.

#### 2.10.3. Trypan Blue Assay

In six-well plates, HeLa cells (5 × 10^5^ cells) were sown, and they were allowed to develop overnight in MEM with 10% FBS added for cell attachment. After that, the medium was changed to MEM with different concentrations of acetone extract (5–100 mg/mL) and incubated for 24 h. The medium was taken out after the necessary incubation time, trypan blue stain (0.4% *w*/*v*) was added to each tube, and the results were examined using inverted microscopy [22].

#### 2.10.4. Brine Shrimp Lethality Test (BSLT)

A brine shrimp lethality assay was conducted to evaluate the cytotoxic potential of various plant extracts [23]. To simulate seawater, 38 g of NaCl was dissolved in 1000 mL of distilled water along with NaOH to maintain a stable pH. Brine shrimp eggs were hatched in this artificial saltwater to produce nauplii. The test samples were prepared by gradually diluting DMSO to concentrations ranging from 25 to 100 *μ*g/mL. Colchicine was used as the reference medication at similar concentrations, while DMSO served as the negative control. At room temperature (25°C), nauplii were visually counted and placed into vials containing 5 mL of simulated saltwater. The samples were then dispensed into premarked vials using a micropipette. After 24 h, the number of surviving nauplii was recorded to assess cytotoxicity.

 $$\begin{array}{c} \%\text{mortality}=\frac{\text{number of nauplii taken}-\text{number of nauplii alive}}{\text{number of nauplii taken}}\times 100. \end{array}$$

### 2.11. Analgesic Activity

#### 2.11.1. Acetic Acid–Induced Writhing Method

The writhing test in mice was conducted using the publication's methodology [24]. In this study, mice were divided into four groups: Group I served as the control and received 1% Tween 80 (1 mL/kg, i.p.), Group II was the standard group treated with Diclofenac Na (10 mg/kg), and Groups III and IV were the test groups administered AERM at doses of 200 and 400 mg/kg body weight, respectively. Twenty mice were randomly assigned to four groups, with five mice in each group. According to the grouping, each animal received its designated treatment, followed by an intraperitoneal injection of 0.6% acetic acid (10 mL/kg, i.p.) within 1 h. After a 5-min latency period, the analgesic effect of the plant extract was evaluated by counting the number of writhing responses—characterized by abdominal muscle contractions and hind limb extension—over 20 min. A decrease in the number of writhes compared to the control group was interpreted as an indication of the extract's analgesic activity and was calculated as the percentage of writhing inhibition using the following formula [25].

 $$\begin{array}{c} \%\text{inhibition}=\frac{\text{Mc}-\text{Mt}}{\text{Mc}}\times 100, \end{array}$$

where Mc is the mean writhes in the control group and Mt is the mean writhes in the test group.

#### 2.11.2. Formalin-Induced Paw Licking Test

The experimental procedure for the formalin-induced licking test was adapted from previously published methods [26]. In this study, mice were divided into four groups: Group I served as the control and received 1% Tween 80 (1 mL/kg, i.p.), Group II was the standard group treated with morphine sulfate (10 mg/kg), and Groups III and IV were the test groups administered AERM at doses of 200 and 400 mg/kg body weight, respectively. Thirty minutes after treatment, each mouse received a subcutaneous injection of 50 *μ*L of freshly prepared 0.6% formalin into the plantar region of the left hind paw, and their behavior was observed for 1 h afterward. The total time spent licking or biting the injected paw, expressed in seconds, was recorded as an indicator of pain response. The antinociceptive effect was evaluated in two phases: the early phase (0–5 min postinjection), representing neurogenic pain, and the late phase (20–30 min postinjection), indicating inflammatory pain [27]. The percentage inhibition of licking and biting time was calculated using a specific formula to assess the analgesic activity of the treatments compared to the control group.

 $$\begin{array}{c} \text{Inhibition }\left(\%\right)=\frac{\text{Lc}-\text{Lt}}{\text{Lc}}\times 100, \end{array}$$

where Lc is the total number of lickings in the control group and Lt is the total number of lickings in the tested group.

### 2.12. In Silico Computer-Aided Virtual Screening

#### 2.12.1. Software Tools

This study used PubChem, DrugBank, MGL Instruments, Protein Data Bank (PDB), AutoDock Vina, UCSF Chimera, and Discovery Studio Visualizer 2020 (BIOVIA) [28].

#### 2.12.2. Ligand Preparation

The selection of these compounds as potential therapeutic agents is primarily determined by their physicochemical and molecular properties, as well as pharmacokinetic parameters such as ADME/T. To assess the viability of the listed compounds as ligands for therapeutic targets, the pKCSM online tool (http://biosig.unimelb.edu.au/pkcsm/) was utilized on September 11, 2024 [29, 30]. Subsequently, their drug-likeness was analyzed using Lipinski's rule of five via the SwissADME web-based platform [31]. The following compounds of the crude acetone extract of *R. maritima* (Figure S1 and Table S1) were used for in silico molecular docking studies: 9,19-cyclo-25,26-epoxyergostan, psi-cholesterol, cholesterol, stigmasta-5,24(28)-dien-3-ol, gamma-sitosterol, phytol, hexadecanoic acid, 2-hydroxy-1, 13-docosenamide, (z), and n-hexadecanoic acid. The molecules were obtained from PubChem in 3D SDF format or converted from 2D to 3D SDF using Open Babel [32]. Ligands were then minimized and exported in pdbqt format using AutoDock tools for docking [33].

#### 2.12.3. Selection of the Target Proteins

Using the PDB of large biological molecules, prospective therapeutic targets for sedation, anxiety, and depression were revalidated after a thorough evaluation of recent studies [34].

#### 2.12.4. Preparation of Target Proteins

The RCSB PDB supplied the following structures: human cytochrome P450 CYP2C9 (PDB: 1OG5), human pancreatic alpha-amylase (PDB: 4W93), putative sugar kinases (PDB: 5ZTJ), tubulin at the colchicine site (PDB: 1SA0), prostaglandin H2 synthase inhibitors (PDB: 1EQG), and cyclooxygenase-2 (prostaglandin synthase-2) (PDB: 4COX). Nonprotein ligands were removed from these structures, and docking simulations were performed using UCSF Chimera and Discovery Studio Visualizer (BIOVIA) to ensure reproducibility [35]. These proteins serve as validated receptors for assessing antioxidant, antidiabetic, antibacterial, cytotoxic, and analgesic activities.

#### 2.12.5. Virtual Filtering

The PyRx-AutoDock Vina software was used for the virtual screening [36].

### 2.13. Statistical Analysis

Microsoft Excel was used for IC_50_ determination and SPSS (Version 25) for ANOVA. “ChemDraw Ultra 12.0.2” was used to draw the chemical structure of the AERM compounds. The data were presented as mean ± SEM, and a post hoc Dunnett test was conducted. The significance level was set at ⁣^∗^*p* < 0.05, ⁣^∗∗^*p* < 0.01, and ⁣^∗∗∗^*p* < 0.001.

## 3. Results

### 3.1. Total Phenolic and Flavonoid Contents

The findings revealed that AERM possesses substantial levels of total phenolic content (TPC) and total flavonoid content (TFC) (Table 1).

### 3.2. Antioxidant Activity

#### 3.2.1. DPPH Radical Scavenging Activity

**Figure 1:**
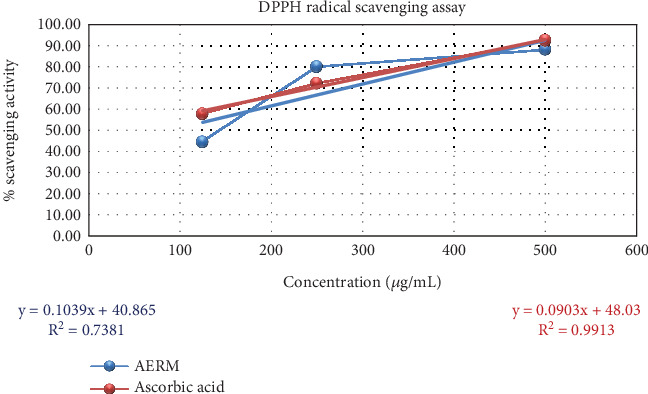
Evaluation of antioxidant activity of AERM through DPPH radical scavenging assay. AERM = acetone extract of *R. maritima*.

By breaking down DPPH, the extract AERM demonstrated potential antioxidant activities of 44.92% ± 5.62%, 80.26% ± 1.67%, and 88.37% ± 0.73% at different concentrations (Figure 1). These results indicate a concentration-dependent increase in antioxidant activity. Additionally, the findings suggest that the extract's antioxidant capacity is comparable to ascorbic acid, a well-known antioxidant standard. The calculated IC_50_ value of the extract against DPPH was 87.92 *μ*g/mL, reflecting its effectiveness in scavenging free radicals. In contrast, ascorbic acid had an IC_50_ value of 21.82 *μ*g/mL.

#### 3.2.2. ABTS Scavenging Activity

**Figure 2:**
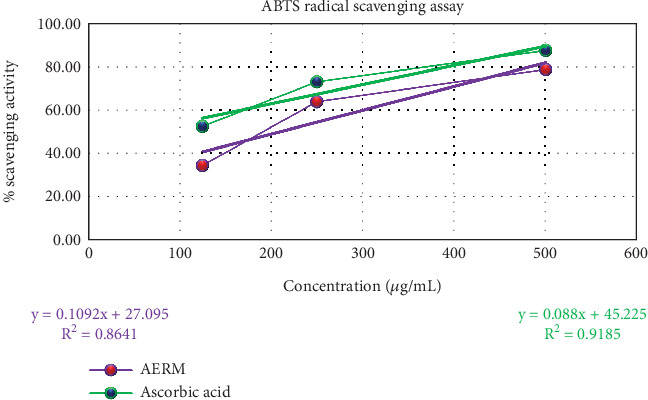
Evaluation of antioxidant activity of AERM through ABTS radical scavenging assay. AERM = acetone extract of *R. maritima*.

Figure 2 illustrates the antioxidant capabilities of the acetone extract of *R. maritima* against free radicals. The extract demonstrated free radical scavenging activities of 34.49% ± 3.54%, 63.77% ± 2.30%, and 78.56% ± 0.86% against ABTS (2,2⁣′-azino-bis(3-ethylbenzothiazoline-6-sulfonic acid)) at different concentrations. These results indicate a concentration-dependent increase in antioxidant activity. The extract AERM exhibited an IC_50_ value of 209.75 *μ*g/mL, reflecting moderate antioxidant activity compared to the standard ascorbic acid, which had an IC_50_ value of 54.26 *μ*g/mL. While the extract showed promising free radical scavenging potential, its activity was less potent than ascorbic acid, suggesting that it may serve as a moderate natural antioxidant source.

### 3.3. Antidiabetic Activity

#### 3.3.1. Alpha-Amylase Inhibition Assay

**Figure 3:**
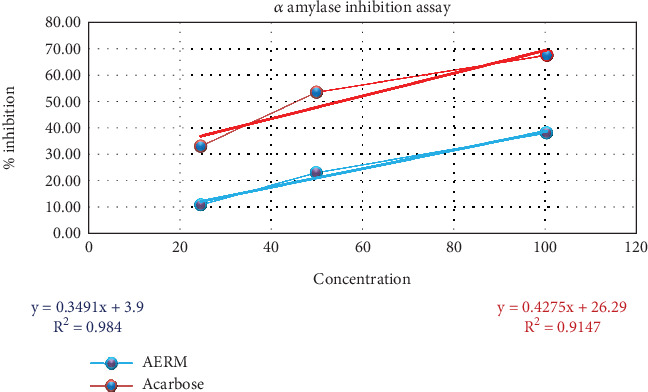
Evaluation of antidiabetic activity of AERM through alpha-amylase inhibition assay. AERM = acetone extract of *R. maritima*.

The extract AERM demonstrated *α*-amylase inhibition ranging from 11.34% ± 1.16% to 38.17% ± 0.94% in a dose-dependent manner (25–100 *μ*g). The IC_50_ value for the extract's *α*-amylase inhibition was estimated at 132.05 *μ*g/mL, indicating notable antidiabetic potential. The standard drug acarbose exhibited significantly higher antidiabetic activity, with an IC_50_ value of 55.46 *μ*g/mL (Figure 3). Since the extract showed promising inhibitory effects on *α*-amylase, its activity was comparable to that of acarbose, suggesting that it may be a natural alternative for managing diabetes.

### 3.4. Antibacterial Activity

#### 3.4.1. Disc Diffusion Method

**Figure 4:**
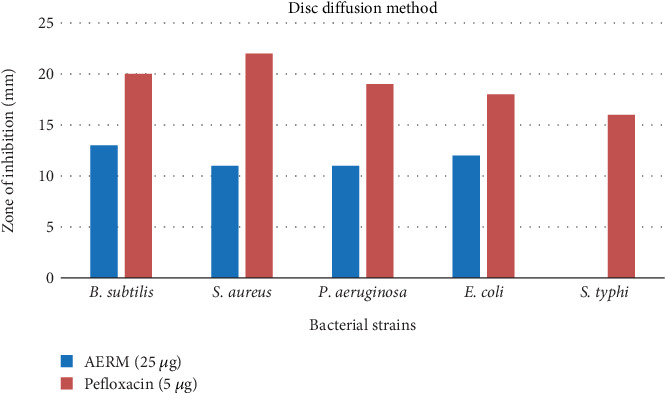
Evaluation of antibacterial activity of AERM against bacterial pathogens. AERM = acetone extract of *R. maritima*.

The antibacterial effectiveness of the test agents was assessed based on their ability to inhibit bacterial growth, as evidenced by the formation of a clear zone of inhibition around the discs. After incubation, the diameters of these inhibition zones were measured in millimeters using a transparent scale, providing a quantitative assessment of the antimicrobial efficacy of the agents. The results are summarized in Table 2 and visually represented in Figure 4. The extract AERM exhibited moderate antibacterial activity against the tested bacterial species, with inhibition zones ranging from 0 to 13 mm. In contrast, the standard antibiotic pefloxacin demonstrated significant antibacterial activity, with inhibition zones ranging from 16 to 22 mm, effective against Gram-positive and Gram-negative bacteria. While AERM showed some potential as an antibacterial agent, its activity was less potent than pefloxacin, highlighting the need for further investigation to enhance its efficacy.

### 3.5. Cytotoxic Activity

#### 3.5.1. Brine Shrimp Lethality Assay

**Figure 5:**
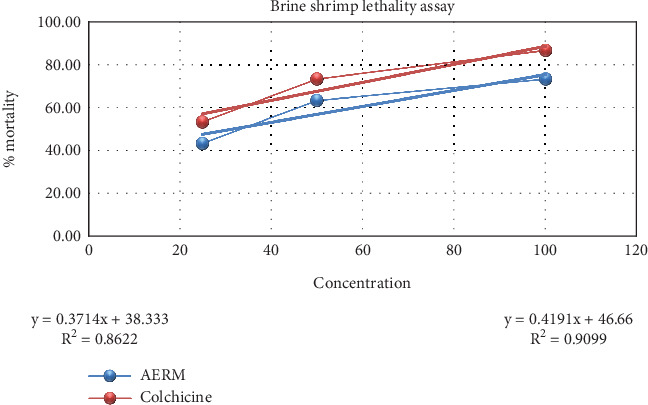
Evaluation of cytotoxic activity of AERM through brine shrimp lethality assay. AERM = acetone extract of *R. maritima*.

The cytotoxic activity of AERM was assessed using the brine shrimp lethality assay. The extract elicited a dose-dependent increase in mortality (%), ranging from 43.33% to 73.33%, at concentrations of 25–100 *μ*g (Figure 5). The extract's LC_50_ value was determined to be 31.41 *μ*g/mL, indicating significant cytotoxic potential. The standard compound colchicine exhibited significantly higher cytotoxicity, with an LC_50_ value of 7.97 *μ*g/mL.

#### 3.5.2. Viability of HeLa Cells by MTT Assay

**Figure 6:**
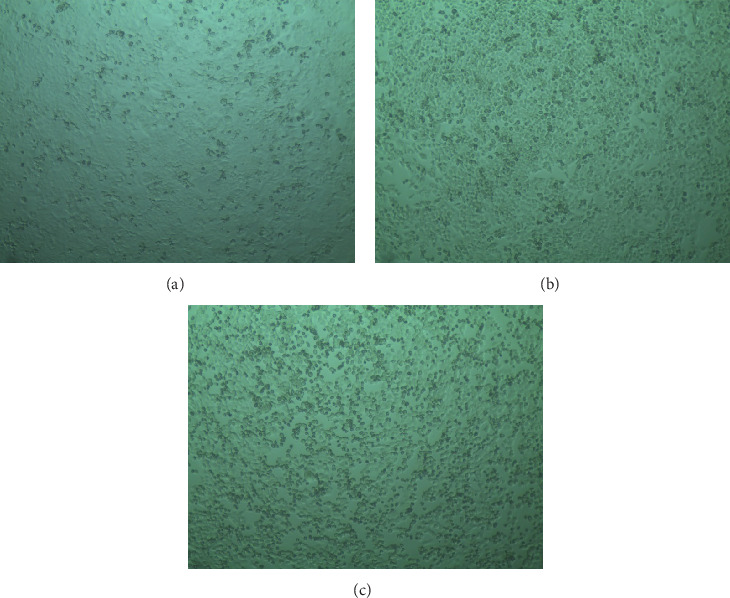
Evaluation of cytotoxic activity of AERM on HeLa cell line through MTT assay. (a) Solvent (−). (b) Solvent (+). (c) AERM. AERM = acetonic extract of *R. maritima*.

When HeLa cells were treated with the acetone extract of *R. maritima*, a concentration-dependent cytotoxic effect was observed. Notably, at a concentration of 100 *μ*g/mL, cell viability was significantly reduced, with a 60%–70% decrease recorded. This drastic decline in cell viability highlights the potent cytotoxic potential of the extract at higher concentrations. These findings suggest that the acetone extract of *R. maritima* may contain bioactive compounds capable of inducing cell death or inhibiting cell proliferation in HeLa cells, warranting further investigation into its mechanism of action and potential therapeutic applications (Table 3 and Figure 6).

### 3.6. Analgesic Activity

#### 3.6.1. Acetic Acid–Induced Writhing Test

**Figure 7:**
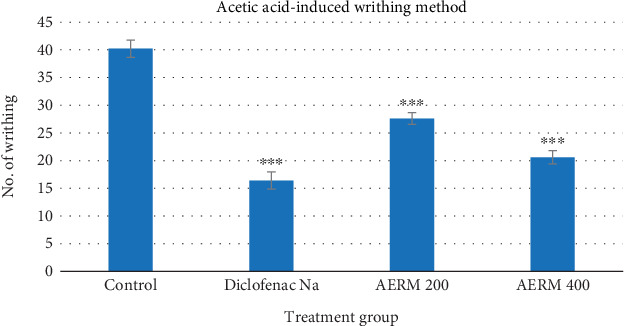
Evaluation of analgesic activity of AERM through the acetic acid–induced writhing method. AERM = acetonic extract of *R. maritima*.

Table 4 illustrates the antinociceptive activity of AERM on acetic acid–induced writhing in mice. AERM produced a statistically significant reduction in the number of writhes (*p* < 0.001) in a dose-dependent manner following intraperitoneal administration. Oral administration of the extract at doses of 200 and 400 mg/kg body weight resulted in a percentage inhibition of 31.34% and 48.75%, respectively. In comparison, the reference medicine, diclofenac sodium, elicited a markedly higher inhibition of 59.2%, indicating superior analgesic efficacy relative to the plant extract (Figure 7).

#### 3.6.2. Formalin-Induced Paw Licking Test

**Figure 8:**
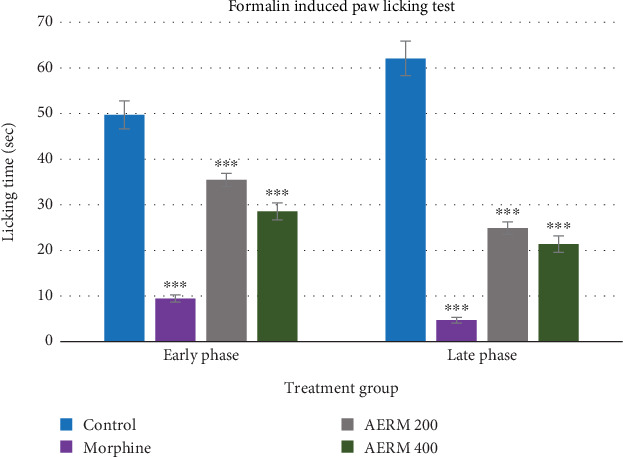
Evaluation of analgesic activity of AERM through the formalin-induced paw licking test. AERM = acetonic extract of *R. maritima*.

The formalin-induced nociceptive assay in mice revealed a dose-dependent attenuation of pain responses, indicating the progressive enhancement of antinociceptive efficacy with increasing doses of the test samples. Morphine sulfate, employed as the reference opioid analgesic at a dose of 10 mg/kg, produced profound antinociceptive effects, yielding 80.99% inhibition during the neurogenic phase and 92.43% during the inflammatory phase. Administration of AERM at 200 mg/kg resulted in a 28.71% inhibition during the early phase, reflecting a relatively modest central analgesic effect. However, the 59.93% inhibition observed in the late phase indicates a more pronounced peripheral antinociceptive activity. Notably, AERM at 400 mg/kg elicited 42.56% inhibition in the early phase and 65.55% inhibition in the late phase, suggesting a dose-dependent enhancement of both central and peripheral analgesic actions, as shown in Table 5 and Figure 8.

### 3.7. In Silico Study

#### 3.7.1. ADME/T and Drug-Likeness Analysis

**Figure 9:**
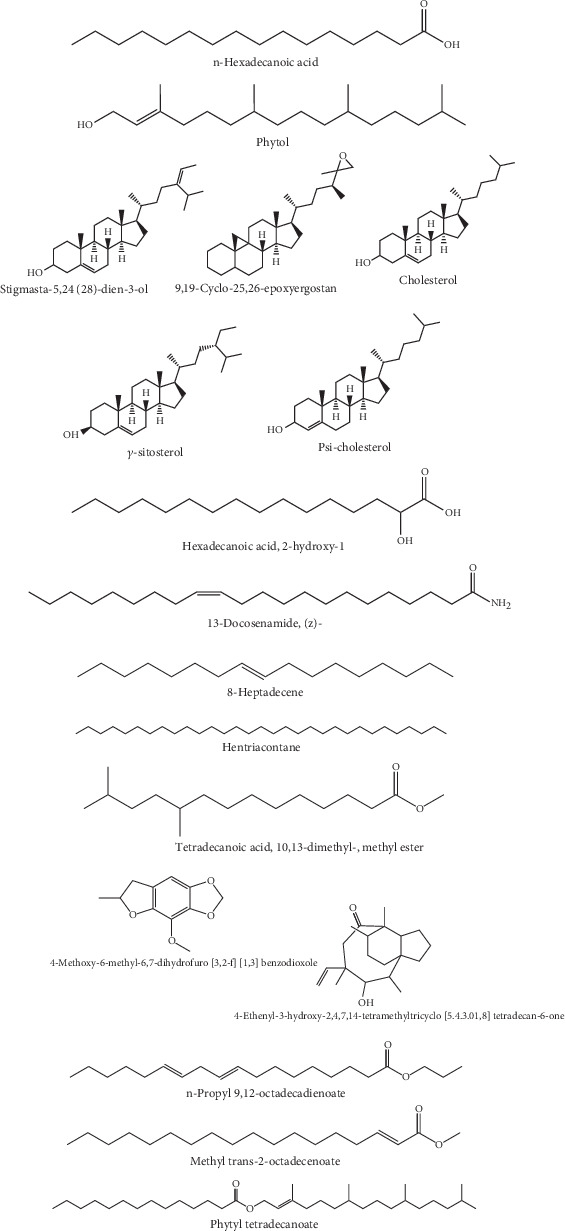
Chemical structures of selected AERM compounds.

The chemical structures of the AERM compounds are given in Figure 9. Before being subjected to docking analysis, the described phytochemicals' pharmacokinetics and drug-likeness were evaluated to identify their therapeutic potential. The pharmacokinetic properties of the discovered substances are shown in Table 6. These profiles may lead to the conclusion that the phytochemicals do not cause mutagenesis or carcinogenesis, and they do not break any of the Lipinski rule requirements. Using the websites pKCSM and SwissADME, in silico ADME/T and drug similarity analysis of compounds discovered in AERM yielded substantial findings.

#### 3.7.2. Molecular Docking for Antioxidant, Antidiabetic, Antibacterial, and Cytotoxic Activities

**Figure 10:**
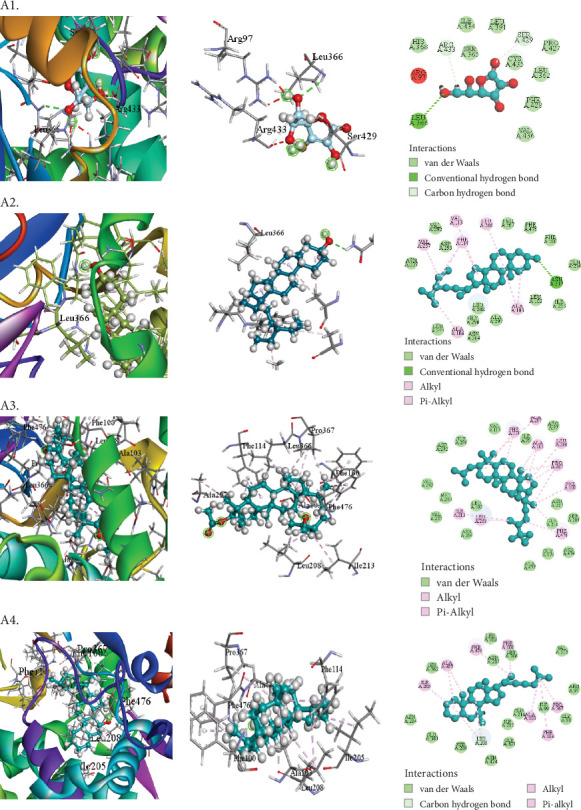
Molecular docking interaction of compounds against the human cytochrome P450 CYP2C9 (PDB: 1OG5): (A1) 9,19-Cyclo-25,26-epoxyergostan. (A2) Psi-cholesterol. (A3) Gamma-sitosterol. (A4) Ascorbic acid (standard).

**Figure 11:**
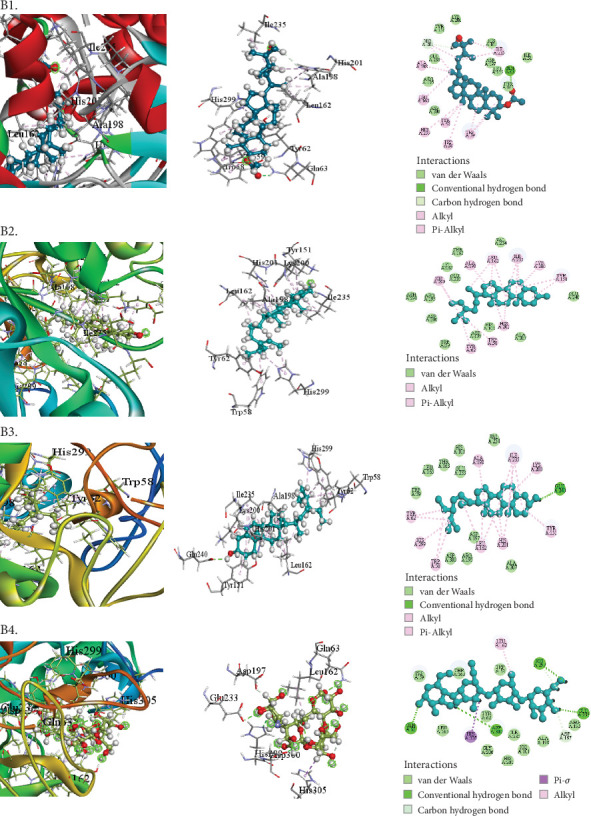
Molecular docking interaction of compounds against the human pancreatic alpha-amylase (PDB: 4W93): (B1) 9,19-Cyclo-25,26-epoxyergostan. (B2) Stigmasta-5,24(28)-dien-3-ol. (B3) Gamma-sitosterol. (B4) Acarbose (standard).

**Figure 12:**
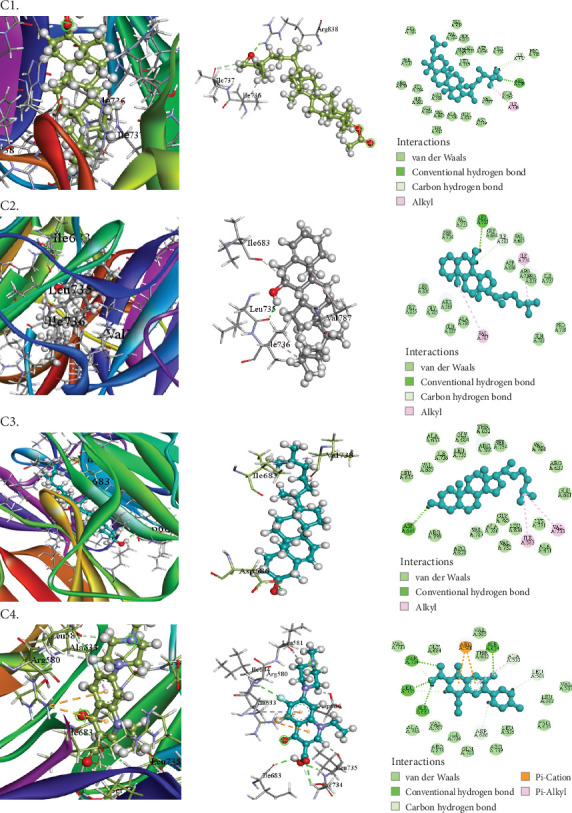
Molecular docking interaction of compounds against the putative sugar kinases (PDB: 5ZTJ): (C1) 9,19-Cyclo-25,26-epoxyergostan. (C2) Psi-cholesterol. (C3) Cholesterol. (C4) Pefloxacin (standard).

**Figure 13:**
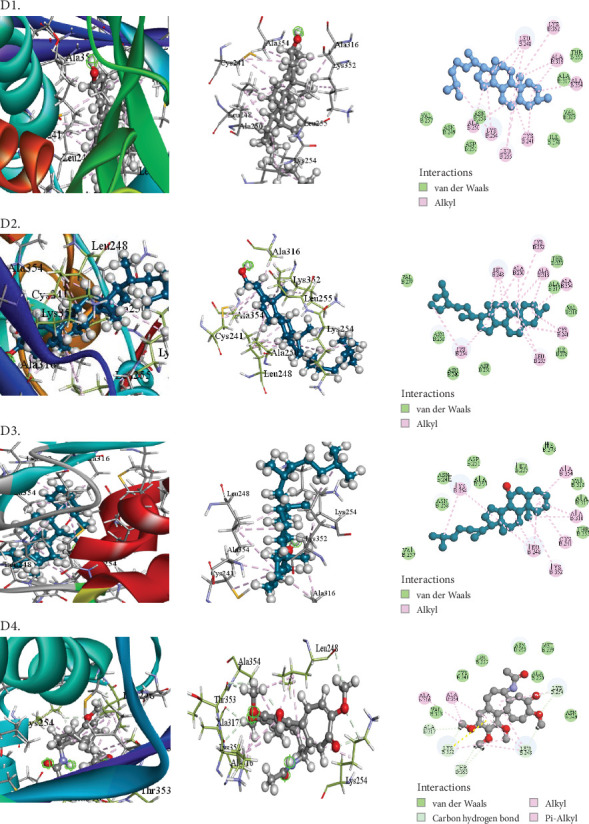
Molecular docking interaction of compounds against the tubulin at colchicine site (PDB: 1SA0): (D1**)** 9,19-Cyclo-25,26-epoxyergostan. (D2) Psi-cholesterol. (D3) Gamma-sitosterol. (D4) Colchicine (standard).

**Figure 14:**
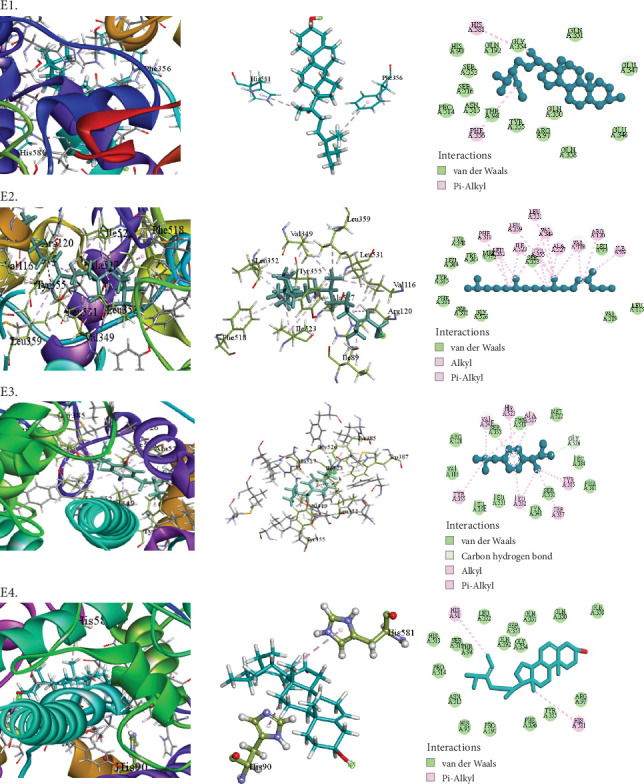
Molecular docking interaction of compounds against the prostaglandin H2 synthase inhibitors (PDB: 1EQG): (E1) Stigmasta-5,24(28)-dien-3-ol. (E2) Gamma-sitosterol. (E3) Phytol. (E4) Ibuprofen (standard).

**Figure 15:**
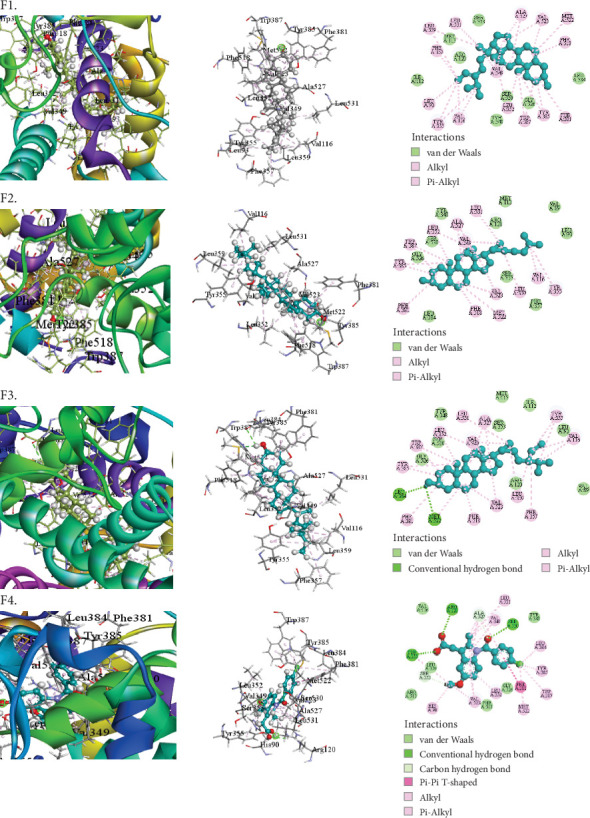
Molecular docking interaction of compounds against the cyclooxygenase-2 (prostaglandin synthase-2) (PDB: 4cox): (F1) Stigmasta-5,24(28)-dien-3-ol. (F2) Cholesterol. (F3) Gamma-sitosterol. (F4) Indomethacin (standard).

The compounds selected from AERM are listed in Table 7, along with their docking scores for antioxidant, antidiabetic, antibacterial, cytotoxic, and analgesic activities against specific protein targets. These targets include tubulin–colchicine:stathmin-like domain complex (PDB ID: 1SA0), human pancreatic alpha-amylase (PDB ID: 4W93), human cytochrome P450 CYP2C9 (PDB ID: 1OG5), crystal structure of GyraseA C-terminal domain (PDB ID: 5ZTJ), prostaglandin H2 synthase inhibitors (PDB ID: 1EQG), and cyclooxygenase-2 inhibitors (PDB ID: 4COX).  Table 8 and Figures 10, 11, 12, 13, 14, and 15 present the docking scores, interaction analyses, and comparisons of the top three AERM compounds with reference drugs for each activity. These comparisons highlight the binding affinities and interactions of the compounds with their respective protein targets, emphasizing their potential pharmacological relevance.

##### 3.7.2.1. Docking Analysis for Antioxidant Activity

The docking analysis results for the antioxidant property are detailed in Table 8. This study utilized human cytochrome P450 CYP2C9 (PDB ID: 1OG5) to identify potential antioxidant compounds from the AERM extract. The binding affinities of the compounds against this protein ranged from −9.7 to −5.5 kcal/mol. Notably, three compounds—9,19-cyclo-25,26-epoxyergostan, psi-cholesterol, and gamma-sitosterol—outperformed the standard inhibitor ascorbic acid (−5.3 kcal/mol), with binding affinities of −9.7, −9.7, and −9.4 kcal/mol, respectively. The top compound, 9,19-cyclo-25,26-epoxyergostan, formed 14 hydrophobic interactions with nine amino acid residues in the active site: ALA103 (2), ALA297, LEU366, PRO367 (3), LEU208, ILE213, PHE100, PHE114 (2), and PHE476 (2) (Table 8 [Section 1] and Figure 10). These interactions highlight the strong binding affinity and potential antioxidant activity of the identified compounds.

##### 3.7.2.2. Docking Analysis for Antidiabetic Activity

All of the substances exhibited a preference for human pancreatic alpha-amylase (PDB ID: 4W93) in terms of antidiabetic properties. Acarbose (−7.2 kcal/mol) had a lower binding score to the target protein than 9,19-cyclo-25,26-epoxyergostan (−9.5 kcal/mol) (Table 8 [Section 2] and Figure 11). The compounds were gamma-sitosterol (−9.3 kcal/mol) and stigmasta-5,24(28)-dien-3-ol (−9.4 kcal/mol). Fourteen hydrophobic contacts with moderate intermolecular distances were generated by 9,19-cyclo-25,26-epoxyergostan. This implies that the medication has a high likelihood of attaching itself to human pancreatic alpha-amylase.

##### 3.7.2.3. Docking Analysis for Antibacterial Activity

9,19-Cyclo-25,26-epoxyergostan exhibited the highest binding affinity (−9.5 kcal/mol) for the crystal structure of GyraseA C-terminal domain (PDB ID: 5ZTJ), surpassing the reference drug pefloxacin (−7.2 kcal/mol). It formed one hydrophobic contact with ILE736, indicating its strong interaction with the target protein. Additionally, other compounds such as psi-cholesterol (−8 kcal/mol) and cholesterol (−7.9 kcal/mol) also demonstrated notable binding affinities (Table 8 [Section 3] and Figure 12). These results highlight the potential of these molecules as effective inhibitors of the crystal structure of GyraseA C-terminal domain, suggesting antibacterial activity.

##### 3.7.2.4. Docking Analysis for Cytotoxic Activity

Each compound demonstrated a strong binding score for the tubulin–colchicine:stathmin-like domain complex (PDB ID: 1SA0) when exhibiting cytotoxic activity. Notably, cholesterol (−7.9 kcal/mol) outperformed the standard colchicine (−7.4 kcal/mol) in terms of binding score to the target protein (Table 8 [Section 4] and Figure 13). The following strongest binders were stigmasta-5,24(28)-dien-3-ol (−7.7 kcal/mol) and psi-cholesterol (−7.6 kcal/mol). Cholesterol formed 15 hydrophobic interactions with key residues in the active site, including CYS241 (2), LEU248 (3), ALA250 (2), LYS254 (2), LEU255 (2), ALA316 (2), ALA354, and LYS352. These interactions occurred at short intermolecular distances, indicating strong binding affinity. This suggests that cholesterol has a high potential to bind effectively to the active site of the tubulin–colchicine:stathmin-like domain complex regulator, contributing to its cytotoxic activity.

##### 3.7.2.5. Docking Analysis for Analgesic Activity

This study screened for potential analgesic compounds using prostaglandin H2 synthase inhibitors (PDB ID: 1EQG) and cyclooxygenase-2 inhibitors (PDB ID: 4COX). The AERM extract compounds showed binding affinities ranging from −7.8 to −2.5 kcal/mol against prostaglandin H2 synthase inhibitors. Notably, stigmasta-5,24(28)-dien-3-ol, gamma-sitosterol, and phytol exhibited binding affinities of −7.8, −7.2, and −7.2 kcal/mol, respectively, which are comparable to the conventional inhibitor ibuprofen (−7.6 kcal/mol). The top compound, stigmasta-5,24(28)-dien-3-ol, formed two hydrophobic interactions with HIS90 and HIS581 in the active site (Table 8 [Section 5] and Figure 14). Additionally, stigmasta-5,24(28)-dien-3-ol demonstrated the highest binding affinity (−10 kcal/mol) to cyclooxygenase-2 inhibitors (PDB ID: 4COX), surpassing the standard drug Indomethacin (−8 kcal/mol). It formed 13 hydrophobic interactions, highlighting its strong binding potential. Other compounds, such as cholesterol (−9.2 kcal/mol) and gamma-sitosterol (−9.2 kcal/mol), also showed notable binding affinities (Table 8 [Section 6] and Figure 15). These results suggest that these compounds have significant potential as analgesic agents through their interactions with key protein targets.

### 3.8. Pass Prediction

The antioxidant, antidiabetic, antibacterial, cytotoxic, and analgesic effects of nine carefully chosen AERM compounds were assessed using the PASS online tool. The findings revealed that compounds with substantial molecular potency had probability of activity (Pa) values greater than probability of inactivity (Pi). The results are given in Table 9.

## 4. Discussion

Ethnomedicine focuses on understanding the traditional medical practices of various ethnic and indigenous communities worldwide that are used to treat specific diseases [37]. The WHO estimates that more than 80% of people globally rely on traditional medicine for their basic healthcare needs [38]. Gathering and preserving the often-unpublished knowledge of medicinal herbs used by different ethnic groups is vital for future healthcare innovations. Furthermore, scientifically validating these traditional remedies is essential for developing effective pharmaceuticals and treatment strategies. The marine environment also serves as a valuable reservoir of bioactive compounds with great promise in creating new functional food components [39]. This study was designed to assess the antioxidant, antidiabetic, antibacterial, cytotoxic, and analgesic properties of acetone extracts from *R. maritima*.

Quantitative analysis revealed that AERM contains 19.04 ± 1.91 mg/g of total phenolics and 14.71 ± 1.09 mg/g of flavonoids. Phenolic compounds are well-known for their pharmacological effects, particularly their antioxidant and antibacterial activities [40]. Furthermore, various studies have highlighted the role of flavonoids and other phenolics in enhancing immune response, neutralizing free radicals, exerting anticancer effects, reducing inflammation, and offering cardiovascular protection [41]. The diverse pharmacological roles of phenolic compounds have been acknowledged, with their antioxidant and antibacterial capabilities being especially noteworthy [40].

The study revealed a significant, dose-dependent decrease in both DPPH and ABTS radical activities upon treatment with the acetone extract of *R. maritima*. The DPPH assay showed maximum radical scavenging activity of 88.37% ± 0.73% at 500 *μ*g/mL, with an IC₅₀ of 87.92 *μ*g/mL. For ABTS, the extract reached a peak activity of 78.56% ± 0.86% at the same concentration, yielding an IC₅₀ of 209.75 *μ*g/mL. These findings confirm the antioxidant potential of the AERM. Oxidative stress, implicated in diseases like cancer, diabetes, cardiovascular and inflammatory conditions, and aging, can be mitigated by antioxidants that neutralize free radicals and prevent lipid peroxidation [42]. In silico docking further validated this antioxidant potential. Three compounds—9,19-cyclo-25,26-epoxyergostan, psi-cholesterol, and gamma-sitosterol—exhibited stronger binding affinities (−9.7, −9.7, and −9.4 kcal/mol, respectively) to CYP2C9 (PDB: 1OG5) than ascorbic acid (−5.3 kcal/mol). Notably, 9,19-cyclo-25,26-epoxyergostan formed 14 hydrophobic bonds with nine amino acids in the active site, suggesting strong interaction and antioxidant efficacy.

The study assessed the antidiabetic effects of *R. maritima* acetone extract (AERM) by testing its in vitro inhibition of *α*-amylase. AERM showed moderate inhibition, with an IC₅₀ of 132.05 *μ*g/mL, compared to 55.46 *μ*g/mL for ascorbic acid. The presence of active phytochemicals like phenols and flavonoids may attribute the antidiabetic properties of the extract [43]. Amylase, a key enzyme in carbohydrate digestion, plays a role in postmeal glucose control and metabolism, making it a valuable target for managing insulin resistance [44]. In silico docking results supported AERM's potential: 9,19-cyclo-25,26-epoxyergostan showed the strongest binding to pancreatic *α*-amylase (−9.5 kcal/mol), outperforming the standard drug acarbose (−7.2 kcal/mol), with 14 hydrophobic contacts, indicating strong affinity.

Experimental investigations revealed that the AERM extract possessed antibacterial properties against five different bacterial strains. The assessment was based on the inhibition zones formed around the discs, which indicated the suppression of bacterial growth. At a concentration of 25 *μ*g/mL, the sponge extract demonstrated antibacterial activity against *Bacillus subtilis* and *Escherichia coli*, comparable to the standard antibiotic pefloxacin (5 *μ*g/mL), highlighting its significant antibacterial potential. However, it showed no activity against *Salmonella* Typhi. The antimicrobial effects are attributed to bioactive constituents such as flavonoids, known for their therapeutic properties [45]. Supporting literature by Modilal et al. [46] and Ranasinghe et al. [47], along with others [48–50], emphasizes the role of such compounds in boosting various bioactivities, including antibacterial, antiviral, and anti-inflammatory properties. Tungmunnithum et al. [41] further demonstrated the effectiveness of flavonoids against diarrhea-causing bacteria. Molecular docking studies reinforced these results: 9,19-cyclo-25,26-epoxyergostan showed the strongest binding to the GyraseA C-terminal domain (PDB ID: 5ZTJ) with a binding energy of −9.5 kcal/mol, exceeding that of pefloxacin (−7.2 kcal/mol), forming a hydrophobic interaction with ILE736. Psi-cholesterol (−8 kcal/mol) and cholesterol (−7.9 kcal/mol) also showed notable binding affinities (Table 8 and Figure 12), suggesting their potential as novel GyraseA inhibitors.

The brine shrimp lethality bioassay is recognized for its versatility in evaluating the cytotoxic and pharmacodynamic activities of both synthetic compounds and plant-derived preparations, including antibacterial, pesticidal, antiviral, and anticancer actions [51, 52]. Among bioassays, the use of brine shrimp nauplii is particularly valued for its simplicity in identifying the cytotoxic properties of crude extracts [53]. The LC_50_ for AERM was calculated at 31.41 *μ*g/mL—substantially higher than the 7.97 *μ*g/mL of the standard drug—indicating its safety margin when administered within therapeutic limits. The lethality is presumably linked to the presence of secondary metabolites [54]. Preliminary phytochemical investigations into *R. maritima* highlighted the presence of flavonoids and phenolic compounds with established anticancer activities [51, 55], thereby justifying the current investigation.

Treatment of HeLa cells with *R. maritima* acetone extract resulted in a concentration-dependent cytotoxic response. At 100 *μ*g/mL, cell viability dropped significantly, by 60%–70%, pointing to the extract's strong bioactivity. This indicates the presence of compounds within the extract capable of inducing cell death or inhibiting proliferation in cancer cells, supporting its potential as a therapeutic agent and warranting further mechanistic studies (Table 3 and Figure 6).

The acetic acid–induced writhing test is a commonly employed method to assess peripheral analgesic properties [56]. The pain generated by acetic acid arises through an indirect process, linked to the increase of prostaglandins PG2 and PG2*α* at receptor sites in visceral tissues. This mechanism implies that carboxylic acid facilitates the release of internal mediators [57]. Experimental animals exhibit writhing behavior due to acetic acid stimulation of chemosensitive nociceptors [58]. NSAIDs act by suppressing sensory neuron responses to these inflammatory agents [59]. The degree of writhing reduction serves as an indicator of analgesic effectiveness [60]. AERM, when administered at 200 and 400 mg/kg doses, significantly decreased the mean writhing count (*p* < 0.001), mirroring the action of standard diclofenac sodium.

The formalin-induced pain model demonstrates a two-phase response: the early neurogenic phase (first 5 min postinjection) and the late inflammatory phase (15–30 min after injection) [51]. Centrally acting analgesics such as opioids are effective in both phases, while NSAIDs primarily target the inflammatory phase [61]. Formalin also induces localized inflammation, visible through swelling and redness, which is mediated by substances like histamine, bradykinin, and serotonin [62]. AERM showed statistically significant analgesic activity at both 200 and 400 mg/kg doses (*p* < 0.05 and *p* < 0.001, respectively). Its efficacy is likely due to the presence of flavonoids and phenolic compounds, which have documented anti-inflammatory and analgesic properties [51, 63].

The PASS online tool was utilized to predict the biological activities of the compounds identified through GC-MS analysis. This tool assigns Pa and Pi scores between 0.000 and 1.000. A compound is considered to have biological potential when its Pa value exceeds its Pi value. Specifically, Pa values below 0.5 indicate low pharmacological activity, values between 0.5 and 0.7 suggest moderate potential, and values greater than 0.7 indicate strong therapeutic efficacy [64]. Several compounds of AERM demonstrated favorable drug-likeness in the PASS prediction results.

Research into the therapeutic potential of *R. maritima* has yielded encouraging results, though important gaps remain. Current data are limited by small sample sizes and insufficient replication, highlighting the need for more statistically sound and rigorously structured studies. Gene expression analysis remains essential for understanding disease-related molecular patterns and linking pharmacodynamic markers to dose-dependent cellular changes. While dose-responsive effects have been observed, further work is needed to pinpoint the optimal therapeutic dosage. Additionally, results from Swiss albino mice may not fully translate to humans, necessitating broader studies across varied animal models.

## 5. Conclusion

The exploration of plant-derived compounds continues to demonstrate their potential in supplementing contemporary medicine, offering therapeutic efficacy coupled with reduced adverse reactions. *R. maritima* has been identified as a viable candidate for pharmacological research. Quantitative phytochemical profiling, along with in vitro, in vivo, and in silico evaluations, indicates the presence of biologically active constituents in AERM with pronounced antioxidant, antidiabetic, antibacterial, cytotoxic, and analgesic properties. Moreover, molecular docking studies reveal high affinities of several compounds for drug target proteins, corroborating experimental findings. To determine its translational potential, extensive preclinical validations followed by clinical evaluations are warranted. Future research should prioritize characterizing AERM's pharmacodynamics and pharmacokinetics within the scope of drug formulation.

## Figures and Tables

**Table 1:** Total phenolic and flavonoid content of AERM. AERM = acetone extract of *R. maritima*.

**Total phenolic content (TPC) of AERM (mg/g)**	**Total flavonoid content (TFC) of AERM (mg/g)**
19.04 ± 1.91 mg/g	14.71 ± 1.09 mg/g

*Note:*Mean ± SEM is used for all values.

**Table 2:** In vitro antibacterial activity of AERM and standard pefloxacin.

**Group**	**Zone of inhibition (mm)**				
	**Gram-positive species**		**Gram-negative species**		
	***B. subtilis***	***S. aureus***	***P. aeruginosa***	***E. coli***	***S.* Typhi**
AERM (25 *μ*g)	13	11	11	12	—
Pefloxacin (5 *μ*g)	20	22	19	18	16

**Table 3:** Cell viability of AERM.

**Sample**	**Survival of cells**
	**HeLa**
Solvent −	100%
Solvent +	> 95%
AERM	60%–70%

**Table 4:** Effect of the acetonic extract of *R. maritima* on acetic acid–induced writhing among mice.

**Groups**	**Treatment**	**Dose and route**	**No. of writhing**	**% of inhibition**
G-I	1% Tween 80 (control)	10 mL/kg; p.o	40.2 ± 1.56	NA
G-II	Diclofenac Na	50 mg/kg; p.o	16.4 ± 1.56^∗∗∗^	59.2
G-III	AERM	200 mg/kg; p.o	27.6 ± 1.07^∗∗∗^	31.34
G-IV	AERM	400 mg/kg; p.o	20.6 ± 1.20^∗∗∗^	48.75

*Note:* Mean ± SEM (*n* = 5). The significance level was set at ⁣^∗∗∗^*p* < 0.001, in comparison to the control.

**Table 5:** Effect of the acetonic extract of *R. maritima* on the formalin-induced paw licking test among mice.

**Groups**	**Treatment**	**Dose and route**	**0–5 min (early phase)**		**20–30 min (late phase)**	
			**Paw licking time(s)**	**% of inhibition**	**Paw licking time**	**% of inhibition**
G-I	1% Tween 80 (control)	10 mL/kg; p.o	49.71 ± 3.06	0	62.07 ± 3.78	0
G-II	Morphine sulfate	10 mg/kg; p.o	9.44 ± 0.79^∗∗∗^	80.99	4.69 ± 0.61^∗∗∗^	92.43
G-III	AERM	200 mg/kg; p.o	35.43 ± 1.43^∗∗∗^	28.71	24.87 ± 1.36^∗∗∗^	59.93
G-IV	AERM	400 mg/kg; p.o	28.55 ± 1.86^∗∗∗^	42.56	21.38 ± 1.81^∗∗∗^	65.55

*Note:* Each value represents the mean ± SEM (*n* = 5).

∗∗∗*p* < 0.001, Dunnett's *t*-test as compared to control.

**Table 6:** In silico AdmetSAR and pharmacokinetic study of chosen substances of AERM. AERM = acetonic extract of *R. maritima*, MW = molecular weight, HBA = hydrogen bond acceptor, HBD = hydrogen bond donor, nRB = number of rotational bonds, TPSA = topological polar surface area, AOT = acute oral toxicity, HIA = human intestinal absorption, HOB = human oral bioavailability, BBB = blood brain barrier, NAT = not AMES toxic, NC = not carcinogenic.

**Compound name**	**Lipinski rules**				**Lipinski's ** **v** **i** **o** **l** **a** **t** **i** **o** **n** ≤ 1	**Veber's rules**		**Toxicity parameters**			**ADME parameters**		
	**M** **W** (**g**/**mol**) < 500	**H** **B** **A** < 10	**H** **B** **D** < 5	**Log ** **p** ≤ 5		**n** **R** **B** ≤ 10	**T** **P** **S** **A** ≤ 140** (**${\overset{{}^{\circ}}{A}}^{2}$**)**	**AMES toxicity**	**Carcinogens**	**AOT**	**HIA**	**HOB**	**BBB**
8-Heptadecene	238.5	0	0	6.65	1	13	0.00	NAT	C	III	0.996	0.55	0.9823
Hentriacontane	436.8	0	0	12.339	1	28	0.00	NAT	C	III	0.9921	0.55	0.9821
4-Methoxy-6-methyl-6,7-dihydrofuro[3,2-f][1,3]benzodioxole	208.2	4	0	1	0	1	36.92	AT	NC	III	0.9908	0.55	0.9087
Tetradecanoic acid, 10,13-dimethyl-, methyl ester	256.42	2	1	5.4082	1	12	37.30	NAT	C	III	0.9842	0.85	0.9788
n-Hexadecanoic acid	256.42	2	1	5.5523	1	14	37.30	NAT	NC	IV	0.9888	0.85	0.9488
n-Propyl 9,12-octadecadienoate	322.5	2	0	6.7531	—	17	26.30	NAT	C	III	0.9972	—	0.9864
Methyl trans-2-octadecenoate	296.5	2	0	6.1969	1	16	26.30	NAT	C	III	0.9952	0.55	0.9818
Phytol	296.5	1	1	6.3641	1	13	20.23	NAT	NC	III	0.9846	0.55	0.9375
Hexadecanoic acid, 2-hydroxy-1	330.5	4	2	4.3641	0	18	66.76	NAT	NC	IV	0.9231	0.55	0.9375
13-Docosenamide, (z)-	337.6	1	1	7.0696	1	19	43.09	NAT	NC	III	1.0000	0.55	0.9972
Cholesterol	386.7	1	1	7.3887	1	5	20.23	NAT	NC	I	1.0000	0.55	0.9749
Psi-cholesterol	386.7	1	1	7.3887	1	5	20.23	NAT	NC	III	1.0000	0.55	0.9832
Gamma-sitosterol	414.7	1	1	8.0248	1	6	20.23	NAT	NC	I	1.0000	0.55	0.9743
Stigmasta-5,24(28)-dien-3-ol	412.7	1	1	7.9449	1	5	20.23	NAT	NC	I	1.0000	0.55	0.9749
4-Ethenyl-3-hydroxy-2,4,7,14-tetramethyltricyclo[5.4.3.01,8]tetradecan-6-one	382.6	4	0	4.3551	0	3	68.82	AT	C	III	1.0000	0.55	0.9533
9,19-Cyclo-25,26-epoxyergostan	498.8	3	0	8.1985	1	7	38.83	NAT	NC	III	0.9966	0.55	0.9478
Phytyl tetradecanoate	506.9	2	0	11.616	2	27	26.30	NAT	C	IV	0.9969	0.17	0.9371

**Table 7:** Binding score of the reported substances of AERM against the human cytochrome P450 CYP2C9 (PDB ID: 1OG5), human pancreatic alpha-amylase (PDB ID: 4W93), crystal structure of GyraseA C-terminal domain (PDB ID: 5ZTJ), and tubulin–colchicine:stathmin-like domain complex (PDB ID: 1SA0), prostaglandin H2 synthase inhibitors (PDB ID: 1EQG), and cyclooxygenase-2 inhibitors (PDB ID: 4COX) for antioxidant, antidiabetic, antibacterial, cytotoxic activity, and analgesic, respectively.

**Compound name**	**PubChem ID**	**Docking score (kcal/mol)**					
		**Antioxidant (1og5)**	**Antidiabetic (4w93)**	**Antibacterial (5ztj)**	**Cytotoxic (1sa0)**	**Analgesic (1eqg)**	**Analgesic (4cox)**
9,19-Cyclo-25,26-epoxyergostan	565753	−9.7	−9.5	−8.4	−7	−2.5	−6.6
Psi-cholesterol	12314669	−9.7	−8.8	−8	−7.6	−6.8	−8.7
Cholesterol	5997	−8.8	−9.1	−7.9	−7.9	−6.9	−9.7
Stigmasta-5,24(28)-dien-3-ol	66739275	−9.2	−9.4	−7.8	−7.7	−7.8	−10
Gamma-sitosterol	457801	−9.4	−9.3	−7.8	−7.5	−7.2	−9.2
Phytol	5280435	−6.6	−5.7	−5	−5.8	−7.2	−7.2
Hexadecanoic acid, 2-hydroxy-1	123409	−6	−5.1	−5	−5.3	−6.4	−6.6
13-Docosenamide, (z)	5365371	−5.6	−5.5	−4.8	−5.8	−6.6	−7.3
n-Hexadecanoic acid	985	−5.5	−5.1	−4.8	−4.8	−6.3	−6.4
**Standards** (ascorbic acid, acarbose, pefloxacin, colchicine, ibuprofen, indomethacin)		−5.3	−7.2	−7.2	−7.4	−7.6	−8

*Note:* This bold value represents the highest score among the compounds.

**Table 8:** AERM's selected phytochemicals in silico binding affinity and nonbonding interaction for antioxidant (1og5), antidiabetic (4w93), antibacterial (5ztj), cytotoxic (1sa0), and analgesic (1eqg and 4cox) properties, respectively.

**Section**	**Protein ID**	**Compound**	**Binding affinity (kcal/mol)**	**Class of bonds**	**Amino acid residue**
1	1OG5	9,19-Cyclo-25,26-epoxyergostan	−9.7	Alkyl	ALA103 (2), ALA297, LEU366, PRO367 (3), LEU208, ILE213
				Pi-alkyl	PHE100, PHE114 (2), PHE476 (2)
		Psi-cholesterol	−9.7	Carbon hydrogen bond	LEU208
				Alkyl	ALA103, ILE205, LEU208 (2), ALA477 (2), PRO367
				Pi-alkyl	PHE100, PHE114, PHE476
		Gamma-sitosterol	−9.4	Conventional hydrogen bond	ASN217
				Alkyl	ALA103 (2), ALA106, VAL113, LEU366, VAL237
				Pi-alkyl	PHE114 (3)
		Ascorbic acid (standard)	−5.3	Conventional hydrogen bond	LEU366
				Carbon hydrogen bond	SER429, ARG433

2	4W93	9,19-Cyclo-25,26-epoxyergostan	−9.5	Conventional hydrogen bond	GLN63
				Carbon hydrogen bond	HIS201
				Alkyl	ALA198, LEU162, ILE235 (2)
				Pi-alkyl	TRP58, TRP59 (5), TYR62, HIS201 (2), HIS299
		Stigmasta-5,24(28)-dien-3-ol	−9.4	Alkyl	LEU162 (3), ALA198, LYS200, ILE235 (3), LEU162
				Pi-alkyl	TRP58, TYR62, TYR151 (2), HIS201 (2), HIS299 (2)
		Gamma-sitosterol	−9.3	Conventional hydrogen bond	GLU240
				Alkyl	LEU162 (3), ILE235 (4), LYS200, ALA198, LEU162
				Pi-alkyl	HIS299, HIS201 (3), TYR151, TYR62 (2), TRP58 (2)
		Acarbose (standard)	−7.2	Conventional hydrogen bond	GLN63, GLU233, HIS299, ASP300
				Carbon hydrogen bond	ASP197, HIS299
				Pi-sigma	HIS305
				Alkyl	LEU162

3	5ZTJ	9,19-Cyclo-25,26-epoxyergostan	−8.4	Conventional hydrogen bond	ARG838
				Carbon hydrogen bond	ILE737 (2)
				Alkyl	ILE737
		Psi-cholesterol	−8	Conventional hydrogen bond	LEU735
				Carbon hydrogen bond	ILE683
				Alkyl	ILE736, VAL736
		Cholesterol	−7.9	Conventional hydrogen bond	LEU735
				Carbon hydrogen bond	ILE683
				Alkyl	VAL787, ILE736
		Pefloxacin (standard)	−7.2	Conventional hydrogen bond	ILE634, SER734, LEU735, ILE683
				Carbon hydrogen bond	ALA633, SER734, LEU735, LEU581, ASP686
				Pi-cation	ARG580 (2)
				Pi-alkyl	ALA633

4	1SA0	Cholesterol	−7.2	Alkyl	CYS241 (2), LEU248 (2), ALA250 (2), LYS254 (2), LEU255 (2), ALA316 (2), ALA354, LYS352
		Stigmasta-5,24(28)-dien-3-ol	−7.7	Alkyl	CYS241 (2), LEU248 (3), ALA250 (2), LYS254 (2), LEU255 (2), ALA316 (2), ALA354, LYS352, LYS352
				Pi-alkyl	HIS90, HIS581
		Psi-cholesterol	−7.6	Alkyl	CYS241, LEU248 (3), LYS254 (2), ALA316 (2), ALA354, LYS352
		Colchicine (standard)	−7.4	Alkyl	LEU248 (2), LYS254, ALA316, ALA354, LYS352
				Pi-alkyl	LEU248, LYS352, ALA354

5	1EQG	Stigmasta-5,24(28)-dien-3-ol	−7.8	Pi-alkyl	HIS90, HIS581
		Gamma-sitosterol	−7.2	Pi-alkyl	PHE356, HIS581
		Phytol	−7.2	Alkyl	VAL116 (2), ARG120 ALA527, VAL349 (2), LEU359 LEU531, ILE523 (2), LEU352, ILE89
				Pi-alkyl	TYR355 (2), PHE518
		Ibuprofen (standard)	−7.6	Carbon hydrogen bond	GLY526
				Alkyl	ALA527, LEU352, VAL349
				Pi-alkyl	TYR355, TYR385, TRP387, VAL349, LEU352, ILE523, ALA527

6	4COX	Stigmasta-5,24(28)-dien-3-ol	−10	Alkyl	LEU93, VAL116, LEU359
				Pi-alkyl	TYR355, PHE357, PHE381, TYR385, TYR387, PHE518
		Cholesterol	−9.7	Alkyl	VAL349 (4), MET522, VAL523 (2), ALA527, LEU352 (2), LEU359, LEU531, VAL116
				Pi-alkyl	TYR355, PHE381, TYR385, TYR38, TRP387 (2), PHE518
		Gamma-sitosterol	−9.2	Conventional hydrogen bond	LEU384, MET522
				Alkyl	VAL349 (4), MET522, VAL523 (2), ALA527 (2), LEU352 (2), LEU359, LEU531, VAL116 (2), LEU359
				Pi-alkyl	TYR355 (2), PHE357, PHE381, TYR385 (2), TYR387 (2), PHE518
		Indomethacin (standard)	−8	Alkyl	LEU531, VAL523, LEU384, MET522
				Pi-alkyl	HIS90, TYR355, TYR385, TRP387 (2), VAL349, VAL523 (2), ALA527 (3), LEU352

**Table 9:** Pass prediction for pharmacologically active compounds of AERM.

**Compounds**	**Biological activity**									
	**Antioxidant**		**Antidiabetic**		**Antibacterial**		**Cytotoxic**		**Analgesic**	
	**Pa**	**Pi**	**Pa**	**Pi**	**Pa**	**Pi**	**Pa**	**Pi**	**Pa**	**Pi**
n-Hexadecanoic acid	0.222	0.045	0.323	0.070	0.300	0.060	0.359	0.039	0.526	0.023
Phytol	0.475	0.008	—	—	0.417	0.026	0.409	0.029	0.300	0.182
Hexadecanoic acid, 2-hydroxy-1	0.297	0.023	0.427	0.011	0.295	0.062	0.431	0.026	0.427	0.088
13-Docosenamide, (z)-	0.167	0.082	0.233	0.091	0.311	0.056	0.247	0.084	0.598	0.008
Cholesterol	0.198	0.056	0.131	0.092	0.267	0.074	0.569	0.014	0.608	0.007
Psi-cholesterol	0.182	0.068	—	—	0.312	0.056	0.502	0.019	0.489	0.043
Gamma-sitosterol	0.178	0.072	—	—	0.283	0.066	0.484	0.021	0.558	0.014
Stigmasta-5,24(28)-dien-3-ol	0.196	0.057	—	—	0.254	0.081	0.497	0.020	0.540	0.018
9,19-Cyclo-25,26-epoxyergostan	0.220	0.045	0.208	0.154	0.232	0.094	0.268	0.073	0.467	0.058

## Data Availability

The raw data supporting the findings of this study will be available from the authors.

## Supporting Information

Supporting Information Additional supporting information can be found online in the Supporting Information section. Figure S1 contains the GC-MS chromatogram of the acetone extract of *R. maritima*, and Table S1 contains compounds identified by GC-MS analysis in the *R. maritima*.

## References

[1] El Omari N., Sayah K., Fettach S. (2019). Evaluation of In Vitro Antioxidant and Antidiabetic Activities of Aristolochia longa Extracts. *Evidence-Based Complementary and Alternative Medicine*.

[2] Dehghan H., Sarrafi Y., Salehi P. (2016). Antioxidant and Antidiabetic Activities of 11 Herbal Plants From Hyrcania Region, Iran. *Journal of Food and Drug Analysis*.

[3] Nair S. S., Kavrekar V., Mishra A. (2013). Evaluation of *In Vitro* Antidiabetic Activity of Selected Plant Extracts. *International Journal of Pharmaceutical Science Invention*.

[4] Ukwubile C. A., Odugu J. A. (2018). Evaluation of Antibacterial and *In Vitro* Antidiabetic Activities of *Phyllanthus amarus* Linn. (*Phyllanthaceae*) Leaf Ethanol Extract. *Journal of Bacteriology & Mycology*.

[5] Egharevba G. O., Dosumu O. O., Oguntoye S. O. (2019). Antidiabetic, Antioxidant and Antimicrobial Activities of Extracts of Tephrosia bracteolata Leaves. *Heliyon*.

[6] Guo J., Jiang X., Tian Y. (2024). Therapeutic Potential of Cinnamon Oil: Chemical Composition, Pharmacological Actions, and Applications. *Pharmaceuticals*.

[7] Maruthanayagam V., Nagarajan M., Sundararaman M. (2013). Cytotoxicity Assessment of Cultivable Marine Cyanobacterial Extracts in *Artemia salina* (Brine Shrimp) Larvae and Cancer Cell Lines. *Toxin Reviews*.

[8] Mohammad M., Islam M. A., Mamun M. J. I. (2025). Methanolic Extract of EdibleLasia spinosaRhizome: A Potential Natural Source of Analgesic, Diuretic, and Thrombolytic Agents. *Journal of Herbs, Spices & Medicinal Plants*.

[9] Vittalrao A. M., Shanbhag T., Kumari M., Bairy K. L., Shenoy S. (2011). Evaluation of Antiinflammatory and Analgesic Activities of Alcoholic Extract of *Kaempferia galanga* in Rats. *Indian Journal of Physiology and Pharmacology*.

[10] La Peyre M. K., Rowe S. (2003). Effects of Salinity Changes on Growth of *Ruppia maritima* L.. *Aquatic Botany*.

[11] Murphy L. R., Kinsey S. T., Durako M. J. (2003). Physiological Effects of Short-Term Salinity Changes on *Ruppia maritima*. *Aquatic Botany*.

[12] Hasle Enerstvedt K., Lundberg A., Jordheim M. (2018). Characterization of Polyphenolic Content in the Aquatic Plants *Ruppia cirrhosa* and *Ruppia maritima*–A Source of Nutritional Natural Products. *Molecules*.

[13] Castro A. J. V., Colares I. G., Franco T. C. R. S., Cutrim M. V. J., Luvizotto-Santos R. (2015). Using a Toxicity Test With *Ruppia maritima* (Linnaeus) to Assess the Effects of Roundup. *Marine Pollution Bulletin*.

[14] Leary S. L., Underwood W., Anthony R. (2013). *AVMA Guidelines for the Euthanasia of Animals: 2013 Edition*.

[15] Shaikh J. R., Patil M. (2020). Qualitative Tests for Preliminary Phytochemical Screening: An Overview. *International Journal of Chemical Studies*.

[16] Islam M. R., Chowdhury M. T., Chowdhury M. M. (2025). Investigating the Secondary Metabolite Profile and Neuropharmacological Activities of *Ipomoea purpurea*: A Multi-Method Approach Using GC–MS, In Vivo, and In Silico Techniques. *Chemistry & Biodiversity*.

[17] Ismail H. F., Hashim Z., Soon W. T., Ab Rahman N. S., Zainudin A. N., Majid F. A. A. (2017). Comparative Study of Herbal Plants on the Phenolic and Flavonoid Content, Antioxidant Activities and Toxicity on Cells and Zebrafish Embryo. *Journal of Traditional and Complementary Medicine*.

[18] Tshibangu P. T., Kapepula P. M., Kapinga M. J. K. (2017). Antiplasmodial Activity of *Heinsia crinita* (Rubiaceae) and Identification of New Iridoids. *Journal of Ethnopharmacology*.

[19] Wan L., Chen C., Xiao Z. (2013). In Vitro and In Vivo Anti-Diabetic Activity of *Swertia kouitchensis* Extract. *Journal of Ethnopharmacology*.

[20] Danish Rizvi S. M., Biswas D., Arif J. M., Zeeshan M. (2011). In-Vitro Antibacterial and Antioxidant Potential of Leaf and Flower Extracts of Vernonia cinerea and Their Phytochemical Constituents. *International Journal of Pharmaceutical Sciences Review and Research*.

[21] Koohpar Z. K., Entezari M., Movafagh A., Hashemi M. (2015). Anticancer Activity of Curcumin on Human Breast Adenocarcinoma: Role of Mcl-1 Gene. *Iranian Journal of Cancer Prevention*.

[22] Zhang X., Wang R., Chen G., Dejean L., Chen Q.-H. (2015). The Effects of Curcumin-Based Compounds on Proliferation and Cell Death in Cervical Cancer Cells. *Anticancer Research*.

[23] Mohammad M., Rasel M. H., Richi F. T. (2025). Neuropharmacological, Cytotoxic, and Anthelmintic Potentials of *Lasia spinosa* (L.) Thwaites Rhizome: In Vivo, In Vitro, and Computational Approach. *Pharmacological Research-Natural Products*.

[24] Balkrishna A., Ranjan R., Sakat S. S. (2019). Evaluation of Polyherbal Ayurvedic Formulation ‘Peedantak Vati’ for Anti-Inflammatory and Analgesic Properties. *Journal of Ethnopharmacology*.

[25] Tadesse T. Y., Berihun Dagnew S., Gobezie Yiblet T., Tesfaw Addis G., Kiflie Z. D. (2024). Evaluation of Anti-Nociceptive and Anti-Inflammatory Activities of Solvent Fraction of the Roots of *Echinops kebericho* Mesfin (Asteraceae) in Mice Model. *Journal of Complementary and Integrative Medicine*.

[26] Froz M. J. L., Barros L. S. P., de Jesus E. N. S. (2024). Lippia alba Essential Oil: A Powerful and Valuable Antinociceptive and Anti-Inflammatory Medicinal Plant From Brazil. *Journal of Ethnopharmacology*.

[27] Dubuisson D., Dennis S. G. (1977). The Formalin Test: A Quantitative Study of the Analgesic Effects of Morphine, Meperidine, and Brain Stem Stimulation in Rats and Cats. *Pain*.

[28] Hossain S., Rabbi S. A. H., Mamun M. J. I. (2025). Antioxidant, Anti-Inflammatory, and Neuropharmacological Potential of *Syngonium podophyllum* Flower Methanolic Extract: Insights From In Vivo, In Vitro, In Silico, and GC–MS/MS Analysis. *Chemistry & Biodiversity*.

[29] Pires D. E. V., Blundell T. L., Ascher D. B. (2015). pkCSM: Predicting Small-Molecule Pharmacokinetic and Toxicity Properties Using Graph-Based Signatures. *Journal of Medicinal Chemistry*.

[30] Mohammad M., Mamun M. J. I., Khatun M. (2025). A Multifaceted Exploration of *Shirakiopsis indica* (Willd) Fruit: Insights Into the Neuropharmacological, Antipyretic, Thrombolytic, and Anthelmintic Attributes of a Mangrove Species. *Drugs and Drug Candidates*.

[31] Daina A., Michielin O., Zoete V. (2017). SwissADME: A Free Web Tool to Evaluate Pharmacokinetics, Drug-Likeness and Medicinal Chemistry Friendliness of Small Molecules. *Scientific Reports*.

[32] Ali M. L., Noushin F., Azme E., Hasan M. M., Hoque N., Metu A. F. (2024). Marine Natural Compounds as Potential CBP Bromodomain Inhibitors for Treating Cancer: An in-Silico Approach Using Molecular Docking, ADMET, Molecular Dynamics Simulations and MM-PBSA Binding Free Energy Calculations. *In Silico Pharmacology*.

[33] Xue Q., Liu X., Russell P. (2022). Evaluation of the Binding Performance of Flavonoids to Estrogen Receptor Alpha by Autodock, Autodock Vina and Surflex-Dock. *Ecotoxicology and Environmental Safety*.

[34] Goodsell D. S., Zardecki C., Di Costanzo L. (2020). RCSB Protein Data Bank: Enabling Biomedical Research and Drug Discovery. *Protein Science*.

[35] Mohammad M., Chowdhury M. T., Eshaque N. H. (2025). Overdose Toxicological Effect of Methanol Extract of Popular Edible Colocasia esculenta Linn. Flowers: Biochemical, Hematological, and Behavioral Study on Swiss Albino Mice. *Food Science & Nutrition*.

[36] Masters L., Eagon S., Heying M. (2020). Evaluation of Consensus Scoring Methods for Auto Dock Vina, Smina and Idock. *Journal of Molecular Graphics & Modelling*.

[37] Bordoloi J., Dihingia A., Kalita J., Manna P. (2020). Ethnomedicinal Plants of North-East India as a Potential Target for Drug Discovery Against Type 2 Diabetes Mellitus. *Advances in Pharmaceutical Biotechnology: Recent Progress and Future Applications*.

[38] Khan M. H., Yadava P. S. (2010). Antidiabetic Plants Used in Thoubal District of Manipur, Northeast India. *Indian Journal of Traditional Knowledge*.

[39] Barbosa M., Valentão P., Andrade P. B. (2014). Bioactive Compounds From Macroalgae in the New Millennium: Implications for Neurodegenerative Diseases. *Marine Drugs*.

[40] Bahri-Sahloul R., Ben Fredj R., Boughalleb N. (2014). Phenolic Composition and Antioxidant and Antimicrobial Activities of Extracts Obtained From *Crataegus azarolus* L. var. *aronia* (Willd.) Batt. Ovaries Calli. *Journal of Botany*.

[41] Tungmunnithum D., Thongboonyou A., Pholboon A., Yangsabai A. (2018). Flavonoids and Other Phenolic Compounds From Medicinal Plants for Pharmaceutical and Medical Aspects: An Overview. *Medicine*.

[42] Marx J. L. (1979). Oxygen Free Radicals Linked to Many Diseases: The Oxygen Free Radicals, Although Made as By-Products of Normal Oxygen-Using Reactions, Nevertheless Have a Wide Potential for Causing Cell Injury. *Science*.

[43] Amalraj S., Mariyammal V., Murugan R., Gurav S. S., Krupa J., Ayyanar M. (2021). Comparative Evaluation on Chemical Composition, In Vitro Antioxidant, Antidiabetic and Antibacterial Activities of Various Solvent Extracts of Dregea volubilis Leaves. *South African Journal of Botany*.

[44] Miura T., Ichiki H., Hashimoto I. (2001). Antidiabetic Activity of a Xanthone Compound, Mangiferin. *Phytomedicine*.

[45] Rathi M. A., Meenakshi P., Gopalakrishnan V. K. (2016). Hepatoprotective Activity of Ethanolic Extract of *Alysicarpus vaginalis* Against Nitrobenzene-Induced Hepatic Damage in Rats. *Journal of Biological Sciences*.

[46] Modilal M. R. D., Anandan R., Sindhu R., Logeshwari M. N. (2015). Screening of Solanum nigrum for Its Phytochemical and Antimicrobial Activity Against Respiratory Tract Pathogens. *International Journal of Pure and Applied Zoology*.

[47] Ranasinghe R., Maduwanthi S. D. T., Marapana R. (2019). Nutritional and Health Benefits of Jackfruit (*Artocarpus heterophyllus* Lam.): A Review. *International Journal of Food Science*.

[48] Mandal P., Babu S. P. S., Mandal N. C. (2005). Antimicrobial Activity of Saponins From *Acacia auriculiformis*. *Fitoterapia*.

[49] Chandrika U. G., Jansz E. R., Warnasuriya N. D. (2005). Analysis of Carotenoids in Ripe Jackfruit (*Artocarpus heterophyllus*) Kernel and Study of Their Bioconversion in Rats. *Journal of the Science of Food and Agriculture*.

[50] Arung E. T., Shimizu K., Kondo R. (2007). Structure–Activity Relationship of Prenyl-Substituted Polyphenols From Artocarpus heterophyllus as Inhibitors of Melanin Biosynthesis in Cultured Melanoma Cells. *Chemistry & Biodiversity*.

[51] Nath A. K., Alam S. S., Ara J. (2025). Evaluation of In Vitro and In Vivo Pharmacological Activity of Elatostema Sessile With In Silico Approaches. *Food Science & Nutrition*.

[52] Meyer B. N., Ferrigni N. R., Putnam J. E., Jacobsen L. B., Nichols D. E. J., McLaughlin J. L. (1982). Brine Shrimp: A Convenient General Bioassay for Active Plant Constituents. *Planta Medica*.

[53] Karchesy Y. M., Kelsey R. G., Constantine G., Karchesy J. J. (2016). Biological Screening of Selected Pacific Northwest Forest Plants Using the Brine Shrimp (*Artemia salina*) Toxicity Bioassay. *Springerplus*.

[54] Özçelik B., Kartal M., Orhan I. (2011). Cytotoxicity, Antiviral and Antimicrobial Activities of Alkaloids, Flavonoids, and Phenolic Acids. *Pharmaceutical Biology*.

[55] Sharifi-Rad J., Seidel V., Izabela M. (2023). Phenolic Compounds as Nrf2 Inhibitors: Potential Applications in Cancer Therapy. *Cell Communication and Signaling*.

[56] Trongsakul S., Panthong A., Kanjanapothi D., Taesotikul T. (2003). The Analgesic, Antipyretic and Anti-Inflammatory Activity of *Diospyros variegata* Kruz.. *Journal of Ethnopharmacology*.

[57] Eva T. A., Mamurat H., Rahat M. H. H., Hossen S. M. M. (2024). Unveiling the Pharmacological Potential of Coelogyne Suaveolens: An Investigation of Its Diverse Pharmacological Activities by In Vivo and Computational Studies. *Food Science & Nutrition*.

[58] Onasanwo S. A., Elegbe R. A. (2009). Anti-Nociceptive and Anti-Inflammatory Properties of the Leaf Extracts of Hedranthera barteri in Rats and Mice. *African Journal of Biomedical Research*.

[59] Akindele A. J., Ibe I. F., Adeyemi O. O. (2012). Analgesic and Antipyretic Activities of *Drymaria cordata* (Linn.) Willd (Caryophyllaceae) Extract. *African Journal of Traditional, Complementary and Alternative Medicines*.

[60] Marchioro M., Blank M. . F. A., Mourão R. H. V., Antoniolli Â. R. (2005). Anti-Nociceptive Activity of the Aqueous Extract of *Erythrina velutina* Leaves. *Fitoterapia*.

[61] Pham H., Spaniol F. (2024). The Efficacy of Non-Steroidal Anti-Inflammatory Drugs in Athletes for Injury Management, Training Response, and Athletic Performance: A Systematic Review. *Sports*.

[62] da Silva Prudêncio R., de Sousa A. K., Silva D. M. M. (2025). Structural Characterization of a Sulfated Polysaccharide From Gracilaria domingensis and Potential Anti-Inflammatory and Antinociceptive Effects. *Carbohydrate Research*.

[63] Shajib M. S., Akter S., Ahmed T., Imam M. Z. (2015). Antinociceptive and Neuropharmacological Activities of Methanol Extract of Phoenix sylvestris Fruit Pulp. *Frontiers in Pharmacology*.

[64] Rakib A., Ahmed S., Islam M. A. (2020). Antipyretic and Hepatoprotective Potential of Tinospora crispa and Investigation of Possible Lead Compounds Through In Silico Approaches. *Food Science & Nutrition*.

