# Supplementary material for: Discovery of Bioactive Properties in the Seagrass Ruppia maritima Extract: A Triplatform Assessment of Antioxidant, Antidiabetic, Antibacterial, Cytotoxic, and Analgesic Activities
Source: ScientificWorldJournal. 2025 Nov 26;2025:5452807. doi: 10.1155/tswj/5452807 (PMC12674862; doi:10.1155/tswj/5452807)
Supplement: Supporting Information — Additional supporting information can be found online in the Supporting Information section. Figure S1 contains the GC-MS chromatogram of the acetone extract of R. maritima, and Table S1 contains compounds identified by GC-MS analysis in the R. maritima. [file 5452807.f1.docx]

**FIGURE S1.** R. maritima acetonic extracts' GC–MS chromatogram.

**Table S1.** 21 compounds identified by GC-MS analysis in R. maritima.

| Compound Name | RT | Area | Area% | Height |
| --- | --- | --- | --- | --- |
| 8-Heptadecene | 9.290 | 6,378.7 | 0.73 | 270,092.0 |
| Hentriacontane | 9.290 | 30,262.5 | 3.46 | 1,391,680.0 |
| 4-methoxy-6-methyl-6,7-dihydrofuro[3,2-f][1,3]benzodioxole | 10.793 | 22,199.1 | 2.54 | 914,682 |
| Tetradecanoic acid, 10,13-dimethyl-, methyl ester | 11.625 | 122,624.1 | 14.00 | 5,391,681.0 |
| [n-Hexadecanoic acid](https://www.ncbi.nlm.nih.gov/pcsubstance/?term=%22n-Hexadecanoic%20acid%22%5bCompleteSynonym%5d%20AND%20985%5bStandardizedCID%5d) | 12.022 | 69,512.6 | 7.94 | 1,508,828.0 |
| [n-Propyl 9,12-octadecadienoate](https://www.ncbi.nlm.nih.gov/pcsubstance/?term=%22n-Propyl%209%2C12-octadecadienoate%22%5bCompleteSynonym%5d%20AND%209949088%5bStandardizedCID%5d) | 13.227 | 28,510.4 | 3.26 | 1,114,945.0 |
| [Methyl trans-2-octadecenoate](https://www.ncbi.nlm.nih.gov/pcsubstance/?term=%22methyl%20trans-2-octadecenoate%22%5bCompleteSynonym%5d%20AND%205370350%5bStandardizedCID%5d) | 13.289 | 30,387.0 | 3.47 | 1,227,543.0 |
| Phytol | 13.388 | 190,105.8 | 21.71 | 7,433,960.0 |
| Hentriacontane | 13.525 | 25,259.8 | 2.88 | 1,100,002.0 |
| Hentriacontane | 15.040 | 50,387.7 | 5.75 | 1,721,554.0 |
| Hexadecanoic acid, 2-hydroxy-1 | 16.654 | 24,156.0 | 2.76 | 1,029,392.0 |
| Hentriacontane | 16.754 | 11,718.3 | 1.34 | 304,880.0 |
| 13-Docosenamide, (z)- | 18.157 | 43,557.1 | 4.97 | 1,148,502.0 |
| Cholesterol | 18.741 | 19,254.0 | 2.20 | 685,186.0 |
| Psi-cholesterol | 21.870 | 21,007.4 | 2.40 | 577,942.0 |
| Gamma-Sitosterol | 22.454 | 76,920.7 | 8.78 | 2,384,549.0 |
| [Stigmasta-5,24(28)-dien-3-ol](https://pubchem.ncbi.nlm.nih.gov/compound/66739275) | 22.553 | 9,502.1 | 1.09 | 302,984.0 |
| 4-ethenyl-3-hydroxy-2,4,7,14-tetramethyltricyclo[5.4.3.01,8]tetradecan-6-one | 23.038 | 7.792.1 | 0.89 | 215,155.0 |
| 9,19-Cyclo-25,26-epoxyergostan | 23.497 | 7,935.1 | 0.91 | 179,533.0 |
| Phytyl tetradecanoate | 24.193 | 16,269.5 | 1.86 | 286,794.0 |
| Totals |  | 875,685.8 | 100.0 | 31,449,211.0 |
